# Ewing's Sarcoma (Primitive Neuroectodermal Tumor) of Seminal Vesicles: A Case Report

**DOI:** 10.7759/cureus.21993

**Published:** 2022-02-07

**Authors:** Ahmed M Badheeb, Anwar A Alshukami, Galal A Bashanfer, Omar M Alkhanbashi, Mohamed A Badheeb

**Affiliations:** 1 Oncology Center, King Khalid Hospital, Najran, SAU; 2 Medicine, Hadhramout University, Mukalla, YEM; 3 Diagnostic Radiology, King Khalid Hospital, Najran, SAU; 4 Pathology and Laboratory Medicine, King Khalid Hospital, Najran, SAU; 5 Urology, King Khalid Hospital, Najran, SAU; 6 General Medicine, King Khalid Hospital, Najran, SAU

**Keywords:** extra-skeletal ewing sarcoma, ewing sarcoma (es), primitive neuroectodermal tumor (pnet), seminal vesicle, seminal vesicle ewing sarcoma

## Abstract

Malignant tumors of the seminal vesicles are rare; they may be of epithelial or mesenchymal origin. Carcinomas are the most common and pelvic sarcoma may be confused with a primary tumor of the seminal vesicles. Little is known of the prognosis and best sequence of treatment in such sarcoma. We report a rare case of extra-skeletal Ewing sarcoma/primitive neuroectodermal tumor of the seminal vesicles in a 54-year-old man who presented with chronic lower abdominal pain, urinary retention, and severe constipation. Pelvic CT scan and MRI confirmed the presence of a soft tissue mass lesion centered on seminal vesicles. A trans-gluteal Tru-cut biopsy confirmed the diagnosis. Three cycles of the preoperative chemotherapy VAC/IE (vincristine, Adriamycin and cyclophosphamide, followed by ifosfamide and etoposide) protocol achieved an excellent clinical response.

## Introduction

Seminal vesicle primary malignant tumors are uncommon. They can arise from either epithelial or mesenchymal cells, with carcinomas being the most prevalent [1]. Ewing's sarcoma of the prostate and seminal vesicle is exceedingly rare in adolescents and young adults representing less than 0.1% of all primary tumors [2,3].

These tumors have a bad prognosis and require aggressive treatment. The secondary malignancy can frequently be seen either from the adjacent organs, the most common of which is the prostate, or as distant metastasis [4]. There is a scarcity of evidence on management regimens, and most treatments are personalized. We describe a case of a 54-year-old man who was effectively treated for a seminal vesicle primitive neuroectodermal tumor (PNET) with chemotherapy and radical surgery.

## Case presentation

**Figure 1:**
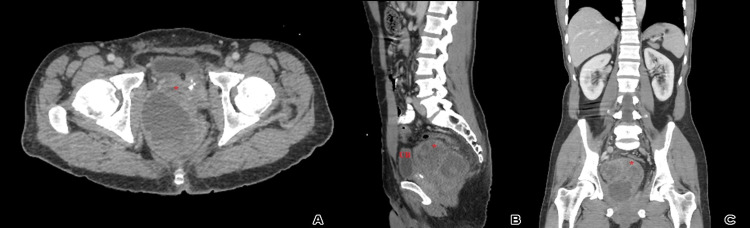
(A) Axial, (B) sagittal and (C) coronal pelvis CT scans with an IV contrast show a well-defined, lobulated, complex, heterogeneously enhanced soft tissue pelvis mass lesion (red asterisk), located between the urinary bladder (UB) and the sacrum with cystic changes, necrosis, and focal effect on the adjacent structures.

A 54-year-old man presented with chronic lower abdominal pain, urine retention, and severe constipation. CT imaging revealed a lobulated, complex, heterogeneously enhanced soft tissue pelvis mass lesion with cystic changes/necrosis, measuring about 11 x 10.2 x 9.8 cm, predominantly located in the floor of the pelvis, between the urinary bladder and the sacrum, likely arising from the seminal vesicles, exerting focal mass effect on the adjacent structures and pelvic floor muscles, with no gross evidence of muscular invasion. The mass was compressing and displacing the adjacent part of the anal canal and rectum to the left side laterally, and the prostate to the left side anterolaterally. Additionally, mild surrounding fat stranding and subcentimetric regional lymph nodes were observed without distant metastasis (Figure 1).

**Figure 2:**
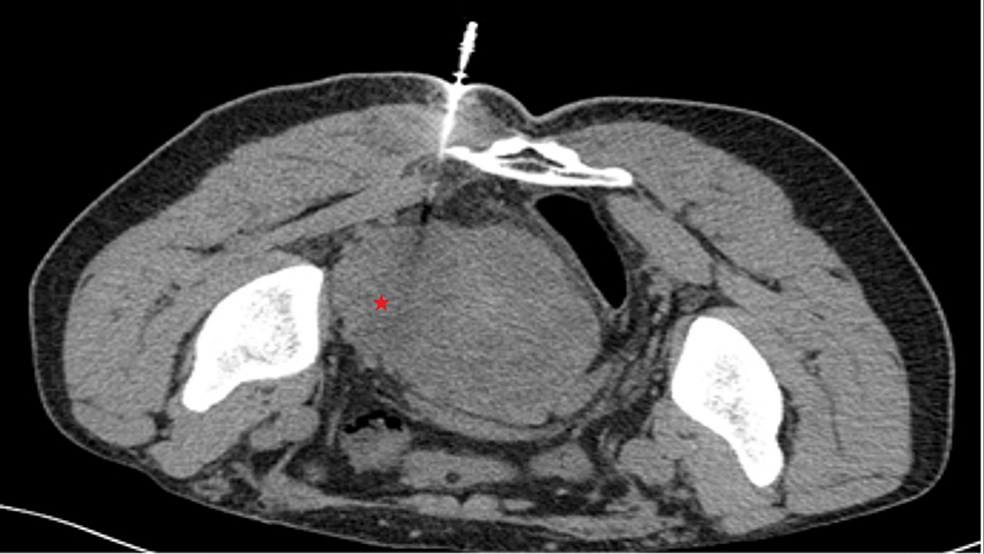
CT-guided core biopsy (Tru-cut), with the red asterisk showing the location of the tumor.

The interventional radiologist had chosen a CT-guided trans-gluteal rather than a trans-rectal and trans-perineal core needle biopsy due to the history of rectal symptoms and the suspected rectal adherence shown on the CT scan (Figure 2).

**Figure 3:**
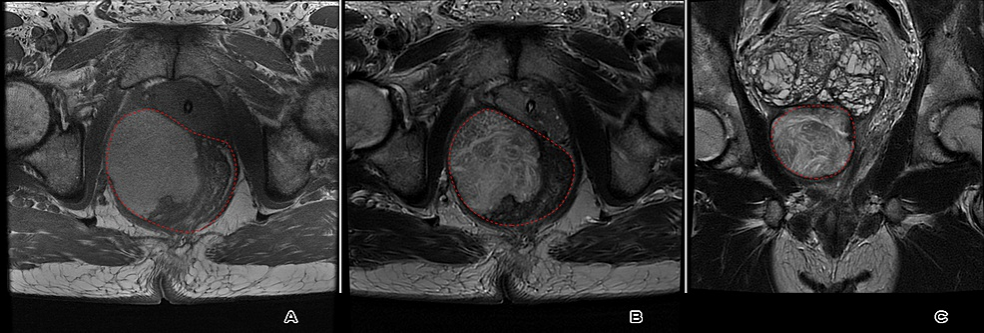
Axial T1WI (A) and T2WI (B) showed a heterogeneous, complex, abnormal hyperintense T1/T2 signal pelvic mass lesion. Coronal T2WI (C) revealed a heterogeneous hyperintense T2 lesion not suppressed on T2 fat saturation images with enlarged seminal vesicles. All are marked by the red dashed line.

**Figure 4:**
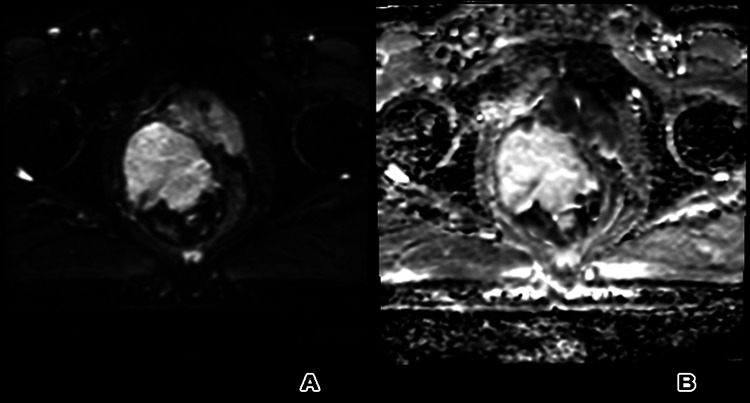
(A) Diffusion-weighted imaging and (B) apparent diffusion coefficient map displayed no restriction.

**Figure 5:**
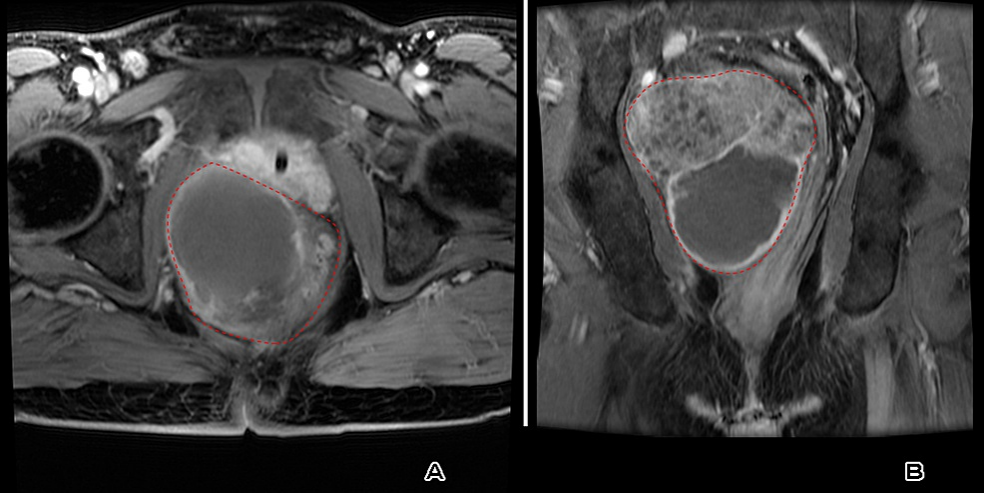
(A) Axial and (B) coronal post-contrast T1 fat saturation image revealed a mixed pattern of enhancement: small, scattered soft tissue nodular enhancement and relatively large non-enhanced areas. All are marked by the red dashed line.

Magnetic resonance imaging of the pelvis confirmed the presence of a well-defined, lobulated, complex, heterogeneous, abnormal-signal-intensity soft tissue mass lesion with a thin hypointense capsule centered on seminal vesicles, and revealed heterogeneous, abnormal, hyperintense T1/T2 signal not suppressed on T2 fat saturation images (of blood/mucinous/proteinaceous contents) (Figure 3) and not restricted on diffusion-weighted images (Figure 4). It showed a mixed pattern of enhancement: small scattered soft tissue nodular enhancement and relatively large non-enhanced areas after IV gadolinium administration (Figure 5). The lesion measured about 9 x 6.6 x 8.2 cm in maximum anterior-posterior, transverse and cranial-caudal dimensions, respectively, was located on the right side of the pelvic floor. Both seminal vesicles were enlarged.

**Figure 6:**
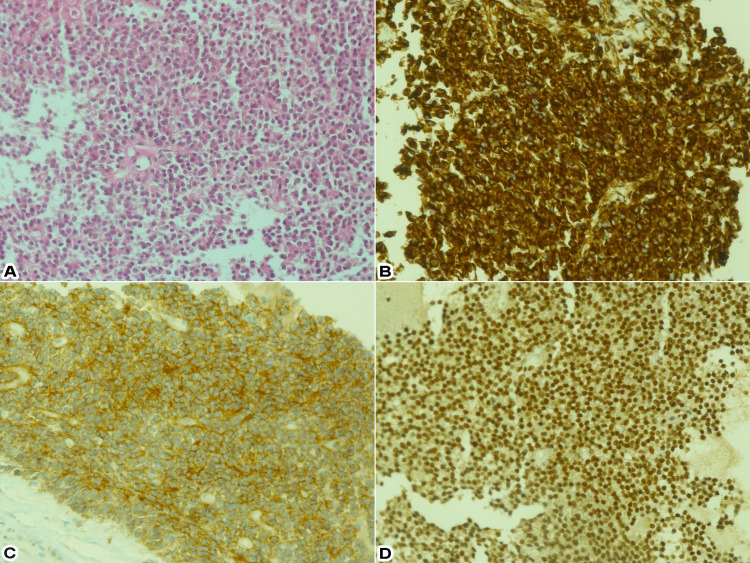
Immunohistochemical analysis using different stains: (A) periodic acid–Schiff (PAS) special stain highlighted the cytoplasmic glycogen, (B) tumor cells positive for vimentin, (C) tumor cells positive for CD99, (D) tumor cells positive for Friend leukemia virus integration-1 (FLI-1).

Histopathological examination revealed proliferated small monotonous round cells. These cells had round nuclei and scant clear cytoplasm and showed positive expression for vimentin, CD99, and Friend leukemia virus integration-1 (FLI-1) immunostains while being negative for cytokeratins, leukocyte common antigen (LCA), chromogranin, neurofilament, prostate-specific antigen (PSA), CD138, desmin, myogenin, CD34, EMA, DOG-1, inhibin, and human melanoma black-45 (HMB-45). Accordingly, immunohistochemical analysis confirmed the diagnosis of a PNET, as shown in Figure 6.

**Figure 7:**
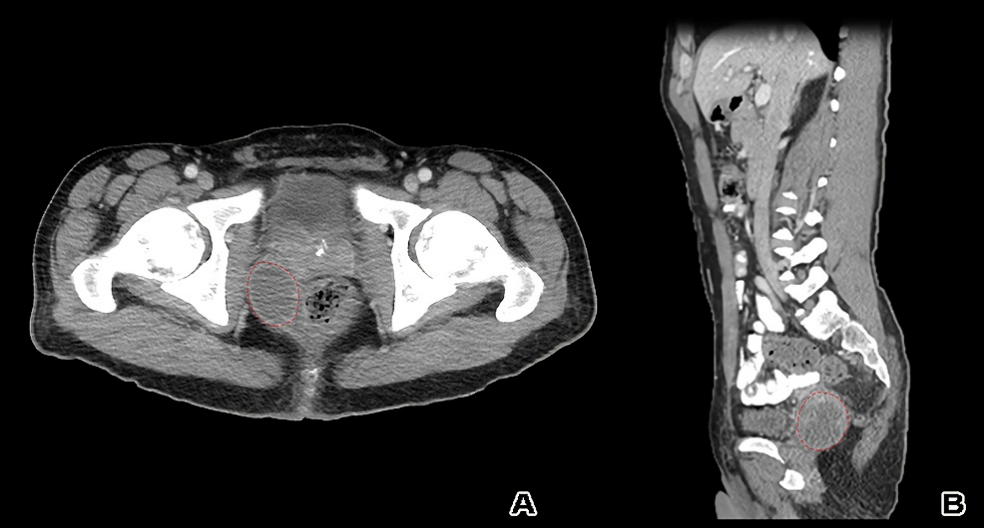
(A) Axial and (B) sagittal CT scan, at the follow-up interval after three cycles of chemotherapy, showed reduction in tumor size (6.5 x 5 x 5 cm) and mass effect but still rectum and prostate gland indentation. The dashed red lines mark the tumor.

**Figure 8:**
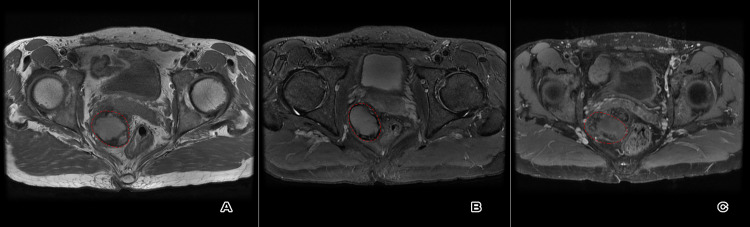
Axial T1 (A), T2 (B) and post-contrast T1 (C) images at the follow-up interval after three cycles of chemotherapy revealing reduction in tumor size and mass effect with residual rectum and prostate gland indentation. The dashed red lines mark the tumor.

A follow-up in two months, after three cycles of induction chemotherapy, revealed almost 50% reduction in the tumor size (6.5 x 5 x 5 cm) and focal mass effect on the adjacent pelvic structures (prostate, rectum, and anal canal (Figures 7, 8).

One month following the third neoadjuvant chemotherapy session, the patient was shifted to another hospital where he underwent robotic excision of the tumor. Surgical pathology showed 70% necrosis with tumor margin focally involving the cauterized peripheral margin. Following the surgery, the patient received concurrent chemoradiotherapy, which was followed by the VAI protocol (VAIA: vincristine, Adriamycin, ifosfamide, without actinomycin D) every three weeks for 14 cycles. The final chemotherapy session was received in December 2020.

## Discussion

PNETs, as a part of the Ewing family, are characterized by small, round blue cells characterized by balanced translocations in chromosome 22. PNETs of seminal vesicle origin are extremely rare, with a few cases documented; in addition, they are more common in children and adolescents, making the age of our patient unusual [5]. The presentation of seminal vesicle PNETs can vary from an incidental finding during imaging to lower abdominal pain, and compressive symptoms such as voiding difficulties, dysuria, urinary frequency or constipation (Table 1) [1-3,4,6].

**Table 1:** Summary of previously published case reports, including tumors' presentation, size, immunostaining and provided therapy

Author	Patient age (years)	Presentation	Size	Immunostaining	Treatment
de Paula et al. (2006) [3]	25	Incidental finding	5 × 4.9 × 5.5 cm	CD-99 and S-100 protein	Chemotherapy with vincristine, cyclophosphamide, ifosfamide etoposide, and doxorubicin, followed by surgery
Lawrentschuk et al. (2008) [4]	26	Lower urinary tract symptoms (irritative)	10.7 cm	CD-99	Chemotherapy with vincristine, cyclophosphamide, ifosfamide, etoposide, Adriamycin, followed by surgery
Ramamurthy et al. (2011) [1]	40	Hematospermia and pain during ejaculation	3 × 4 cm	Cytokeratin, vimentin, CD-99, S-100	En bloc resection of the tumor and ureteric reimplantation
Crestani et al. (2014) [6]	50	Incidental finding	Two masses 8 cm and 3 cm in the widest diameter	CD-99	Surgery followed by adjuvant therapy with vincristine, cyclophosphamide, ifosfamide, etoposide, Adriamycin, and actinomycin D
Berkut et al. (2020) [2]	35	Left quadrant pain	15 × 12 cm	CD-99	Chemotherapy with vincristine, cyclophosphamide, ifosfamide, etoposide, Adriamycin, followed by surgery

The primary localized seminal vesicle tumor is possibly curable; however, the secondary spread, mainly from the prostate, bladder, and rectum, is usually an advanced disease with poorer outcomes; therefore, differentiation between primary and secondary tumors is crucial. Negativity for both PSA and prostate-specific acid phosphatase immunostains is crucial for diagnosing the primary one [7]. Immunostains are essential in establishing the diagnosis of PNETs. CD-99 positivity is a characteristic of Ewing's sarcoma. In addition, negativity for cytokeratins, LCA, chromogranin, neurofilament, PSA, CD138, desmin, myogenin, CD34, EMA, DOG-1, inhibin, and HMB-45 helps in excluding other etiologies [8,9].

Pelvic seminal vesicle PNETs have a significantly poorer five-year survival than non-pelvic ones (34% vs. 57%; p<0.001). Studies from UK that had used ifosfamide-based chemotherapy in Ewing's sarcoma showed that the survival benefit was limited to the non-pelvic cases (p<0.001) [10].

We followed the standard road map in treating Ewing's sarcoma, starting with chemotherapy followed by radical surgery, taking into consideration that Ewing's family of tumors is systemic in nature, with most patients harboring subclinical metastasis upon diagnosis. Local therapy alone ends with a high relapse rate (80%-90%) [11]. Multiagent chemotherapy used before and after the local treatment in localized cases has been noted to have a significant impact on survival. The five-year survival rate is approximately 70%, and the 10-year survival rate is 50% [11-17]. The adoption of a multidisciplinary approach (chemotherapy, surgery, and radiotherapy) has improved the prognosis of Ewing's sarcoma in the last three decades [17].

## Conclusions

Based on our observation, we believe that a multimodality approach starting with preoperative induction chemotherapy is very helpful in the downstaging of the large seminal vesicle Ewing's sarcoma. The standard chemotherapy protocols used in the skeletal Ewing's sarcoma seem to be effective in the extra-skeletal ones. Optimal usage of the immunohistochemical stains can lead to the proper characterization of the tumor origin. Further confirmatory data is needed to support our conclusion.
